# Cost-Effectiveness of a Continuous Glucose Monitoring Mobile App for Patients With Type 2 Diabetes Mellitus: Analysis Simulation

**DOI:** 10.2196/16053

**Published:** 2020-09-17

**Authors:** Shintaro Tsuji, Tomoki Ishikawa, Yasuhiro Morii, Hongjian Zhang, Teppei Suzuki, Takumi Tanikawa, Jun Nakaya, Katsuhiko Ogasawara

**Affiliations:** 1 Graduate School of Health Sciences Hokkaido University Sapporo Japan; 2 Institute for Health Economics and Policy Tokyo Japan; 3 Art and Sports Business Iwamizawa Campus Hokkaido University of Education Hokkaido Japan; 4 Department of Radiological Technology Hokkaido University of Science Sapporo Japan

**Keywords:** Markov model, telehealth, continuous glucose monitoring (CGM), type 2 diabetes mellitus, cost-effectiveness, incremental cost and effective ratio (ICER)

## Abstract

**Background:**

Apps for real-time continuous glucose monitoring (CGM) on smartphones and other devices linked to CGM systems have recently been developed, and such CGM apps are also coming into use in Japan. In comparison with conventional retrospective CGM, the use of CGM apps improves patients’ own blood glucose control, which is expected to help slow the progression of type 2 diabetes mellitus (DM) and prevent complications, but the effect of their introduction on medical costs remains unknown.

**Objective:**

Our objective in this study was to perform an economic appraisal of CGM apps from the viewpoint of assessing public medical costs associated with type 2 DM, using the probability of developing type 2 DM–associated complications, and data on medical costs and utility value to carry out a medical cost simulation using a Markov model in order to ascertain the cost-effectiveness of the apps.

**Methods:**

We developed a Markov model with the transition states of insulin therapy, nephrosis, dialysis, and cardiovascular disease, all of which have a major effect on medical costs, to identify changes in medical costs and utility values resulting from the introduction of a CGM app and calculated the incremental cost-effectiveness ratio (ICER).

**Results:**

The ICER for CGM app use was US $33,039/quality-adjusted life year (QALY).

**Conclusions:**

Sensitivity analyses showed that, with the exception of conditions where the transition probability of insulin therapy, utility value, or increased medical costs increases, the ICER for the introduction of CGM apps was below the threshold of US $43,478/QALY used by the Central Social Insurance Medical Council. Our results provide basic data on the cost-effectiveness of introducing CGM apps, which are currently starting to come into use.

## Introduction

According to a 2016 World Health Organization (WHO) global report of patients with type 2 diabetes mellitus (DM), there are currently over 420 million people with this condition worldwide, a 3.9-fold increase compared with 1980 [1]. In Japan, the number of people with type 2 DM reached 3.29 million in 2017, representing an increase of over 120,000 from the previous survey in 2014 [2]. The increased medical costs of diabetes and related complications associated with the rising number of people with type 2 DM are becoming an issue [3]. The total medical cost of type 2 DM in Japan is US $10.6 billion, and the cost of related diseases is US $38.3 billion, accounting for around 14% of the country's total medical expenditure of US $354.8 billion [4]. The complications of type 2 DM include nephropathy, neuropathy, and retinopathy; nephropathy, in particular, is a driver of increasing medical costs because patients with this condition frequently transition to dialysis, which costs around 5 million yen per year. To prevent medical costs from ballooning further, it will be necessary to both prevent the onset of type 2 DM itself and control its complications, which requires control of elevated blood glucose levels with insulin administration or oral medication. Proactive treatments to lower blood glucose levels, however, are leading to increases in the occurrence of cardiovascular disease and coma as side effects of hypoglycemia [5,6]. In a recent large-scale retrospective study using a medical fees database, around 20,000 patients with type 2 DM were hospitalized annually for hypoglycemia, 3.8% of whom died [7]. In the same analysis, the estimated incidence of hypoglycemia in patients with type 2 DM was 2.1 episodes/1000 people/year and was associated with incidents such as traffic accidents and falls. Preventing the progression of type 2 DM and its associated complications thus requires treatments to control hyperglycemia while avoiding putting patients in a hypoglycemic state, and this necessitates the daily real-time assessment of blood glucose levels by individual patients. One current method of hypoglycemia management for patients with type 2 DM is retrospective continuous glucose monitoring (CGM), in which blood glucose measurements over a certain period are aggregated and the user examines them after the event to determine any trends [8-10], but this method entails measuring blood glucose levels at regular intervals, and checking blood glucose levels in real time and taking measures to avert hypoglycemia or other conditions are difficult tasks. To resolve this issue, apps for real-time CGM on smartphones and other devices linked to CGM systems have recently been developed, and such CGM apps are also coming into use in Japan. CGM apps enable medical staff in distant locations and patients' families to monitor and share blood glucose level data via smartphones or other mobile devices. Clinical trials are currently underway to investigate the clinical effectiveness of CGM apps in actual use, such as whether they reduce the incidence of complications or have any effect on HbA1c levels, and it is hoped that, in comparison with conventional retrospective CGM, they may help delay the progression of type 2 DM and control complications by improving patients’ own blood glucose control, enabling real-time patient guidance and family members to monitor patients’ blood glucose levels. In advance of their introduction, however, simulations to establish their effect on medical costs will also be required. Our objective in this study was to carry out an economic evaluation of CGM apps from the viewpoint of assessing public medical costs associated with type 2 DM, using a Markov model of the probability of transitioning to type 2 DM–associated complications and data on medical costs and utility value to carry out a medical cost simulation in order to ascertain the cost-effectiveness of the apps.

## Methods

### Analysis Design

The subjects of this study were patients with type 2 DM receiving insulin therapy, and changes in their medical costs and utility values as the result of the introduction of a CGM app were estimated using a Markov model. In general, there are several modeling designs to measure or simulate cost-effectiveness, of which the most common are the Markov and decision tree models. The latter is used in situations where cost-effectiveness of multiple treatments are evaluated, when the patient condition changes irreversibly, or with a relatively short time period, while the Markov model is more suitable for diseases that span longer time periods, such as chronic diseases [11]. The duration of DM treatments is generally long, and the patient condition can change multiple times. For these reasons, the Markov model was more suitable than the decision tree model for this study.

**Figure 1:**
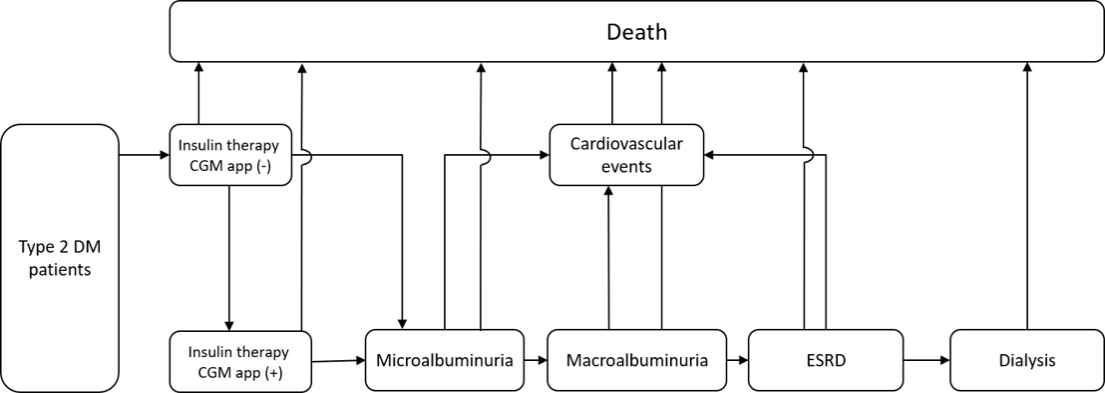
Overview of the Markov model. CGM: continuous glucose monitoring; DM: diabetes mellitus; ESRD: end-stage renal disease.

The main patient states in the constructed model were diabetic nephropathy, dialysis, and cardiovascular disease, all of which have major effects on the medical costs of patients undergoing insulin treatment. To simulate this in a more detailed way, the patient’s disease situation was classified as any of following; insulin therapy, microalbuminuria, macroalbuminuria, end-stage renal disease (ESRD) dialysis, cardiovascular events, or death. The assumption was that the first complication developed by patients receiving insulin therapy would be diabetic nephropathy, followed by either cardiovascular disease induced by type 2 DM or the introduction of dialysis. It was also assumed that diabetic nephropathy would progress to the point at which dialysis was required. The model also assumed that patients with diabetic nephropathy, with cardiovascular disease, or undergoing dialysis would eventually die. The Markov model was updated at yearly intervals for a 20-year analysis period (Figure 1).

The Markov model was adopted to define the patient’s chronic condition at any point in time. The construction of the model is based on the Markov property that the state at any time point is dependent only on the state at the previous time point. If the initial number of patients used in the model is *X,* the model is updated at 1-year intervals, and the total time period is *n* years, the medical costs of state *k* (*k* = *j*_1_, *j*_2_, *j*_3_, ... *j*_n_) in year *t* (===*t* = 1, 2, 3, ... *n*) can be expressed by Equation (1), where the transition probability is *p*_(*jt,jt*-1)_ = *P*(*k_t_* = *j_t_* | *k_t_*_-1_= *j_t_*_-1_) and the discount rate on yearly medical costs is 4%, with C_kt_ = C_t-1_/(1+0.04)^t^ [8]. Similarly, the total medical costs in the model can be expressed as the cumulative sum for the patient states over the number of years concerned.

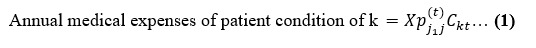



### Initial Sample Size

We initially included 1.2 million patients identified as undergoing insulin therapy from open data held in the NDB (the Japanese national database of health insurance claims and specific health checkup data) published by the Ministry of Health, Labour and Welfare (MHLW) [12]; after the number of patients was adjusted for the number of times a claim was made for a “blood glucose self-measuring instrument,” the final sample size included 686,000 patients judged to be capable of using a CGM, who had not developed nephrosis [13], who were able to use a smartphone [14], and who had not had any skin problems caused by sensor-induced itching [15]. Surveys have indicated that the rate of use of health care apps is approximately 10% [16], and it was assumed that this will tend to increase in the future, so the transition probability that patients will start using the app was set to correspond to 15% of the patients in the study over the 20-year period assumed by the model, equivalent to 100,000 users.

### Assignment of Transition Probabilities in the CGM App Non-Use Model

The transition probability that patients receiving insulin therapy would develop diabetic nephropathy was assigned with reference to the results of the Kumamoto Study [13] of type 2 DM patients in Japan. The transition probability that patients with diabetic nephropathy would develop cardiovascular disease induced by type 2 DM was defined using results of the United Kingdom Prospective Diabetes Study [17]. The ultimate transition probability from diabetic nephropathy to death was defined using the Japan Society for Dialysis Therapy Annual Report [18], and the transition probability from cardiovascular disease induced by type 2 DM to death was defined with reference to the numbers of deaths from different diseases published by the MHLW [19]. This shows the transition probabilities between the different states for patients not using a CGM app (upper part of Figure 1). We treated the transition probabilities in the models as constant values during the time horizon.

### Assignment of Transition Probabilities in the CGM App Use Model

The probability that patients undergoing insulin therapy while using a CGM app was defined as 2.6% of the initial patient population described previously. The transition probability that patients receiving insulin therapy while using any CGM app would develop diabetic nephropathy was obtained from the rate of onset of patients in the Kumamoto Study [13], which is a randomized prospective 6-year study of complications of type 2 DM. The other transition probabilities for patients using a CGM app were assumed to be equivalent to those of patients not using such an app. Sensitivity analyses were conducted to investigate the effects of the probabilities so as to exclude arbitrariness of value selection at this point.

### Validity of the Models

Finally, to verify the validity of the Markov model for CGM app non-use, clinical data not used in the development of the model were used to verify the estimated medical costs. This shows the transition probabilities between the different states for patients using a CGM app (lower part of Figure 1).

### Assignment of Quality-Adjusted Life Years (QALY)

The index of effectiveness used in the cost-effectiveness analysis performed in this study was quality adjusted life years (QALY). QALY is an indicator of survival that takes quality of life into account. The utility value was set at 1 for good health and 0 for death, with states under illness expressed as numbers between 0 and 1. Utility values can be measured by means such as quality of life surveys.

**Table 1:** Probability value of each patient condition.

Transition (to each condition)		Probability value, %		References	
Insulin therapy		2.60		National Health and Nutrition Survey [20]	
**Microalbuminuria**					
	App use group		4.8		Statistics of Medical Care Activities in Public Health Insurance [21]
	App non-use group		1.6		Statistics of Medical Care Activities in Public Health Insurance [21]
Macroalbuminuria		2.80		United Kingdom Prospective Diabetes Study 64 [17]	
Dialysis		2.30		Patient surveys [22]	
Cardiovascular events		10.0		Viana et al [23]	
Death		12.3		Patient surveys [22]	

In this study, we used the utility value measured for type 2 DM patients in Japan. When they could not be obtained from the literature, the utility values for patients with complications were analyzed as the range of variation in the sensitivity analysis ±10% so as to grasp the range of uncertainty, which at the same time excludes arbitrariness in the setting of each parameters. For example, the utility values for the different stages of diabetic nephropathy, one of the conditions addressed in this study, comprising microalbuminuria, macroalbuminuria, and ESRD, were assumed to be values for diabetic nephropathy during insulin therapy without taking the stage classification by Sakamaki et al [24] into account. The utility values of diabetic nephropathy in patients undergoing insulin treatment in combination with either a cardiovascular event or dialysis were taken from the literature, as shown in Table 1. A 4% discount rate was applied to utility values.

### Assignment of Medical Costs

To start with, values for the medical costs of insulin therapy, microalbuminuria, macroalbuminuria, ESRD, cardiovascular disease (induced by type 2 DM), and dialysis were taken from those used in previous studies [25-30] (Table 2). The medical cost of a CGM app was calculated as the cost of a CGM sensor (US $57.39 [6,600 yen] × 3) and transmitter (US $761.74 [87,600 yen]), with the cost of installing and using the app on a mobile device assumed to be free. The medical costs of the various complications, such as developing ESRD while undergoing insulin therapy, were calculated by combining the costs of the different states, as shown in Table 3. The medical costs used in this study were based on outpatient treatment.

To calculate the changes in medical costs for each disease state, the yearly medical costs and the yearly transition probabilities were multiplied by the initial numbers of patients, and the values were aggregated over a 20-year period. Medical expenses estimated from the Markov model for *n* years were calculated using Equation (2). In this equation, all indices are the same as in Equation (1).

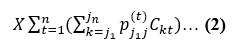



**Table 2:** Utility value of each patient condition.

Patient condition	Utility value	References
Insulin therapy	0.83 (0.79-0.88)	Sakamaki et al [24]
Insulin therapy + diabetic nephropathy	0.81 (0.72-0.90)	Sakamaki et al [24]
Microalbuminuria	0.81	Sakamaki et al [24], Okubo et al [31]
Macroalbuminuria	0.81	Sakamaki et al [24], Okubo et al [31]
End-stage renal disease	0.81	Sakamaki et al [24], Okubo et al [31]
Cardiovascular events	0.71	Hara et al [25]
Diabetic nephropathy	0.68	Takura et al [26]

**Table 3:** Medical fee for each patient status.

Patient condition	Annual medical expenses, US $ (yen)	References
Insulin therapy	4,891.76 (562,552)	Ministry of Internal Affairs and Communication [14], Wong et al [15], Dentsu Digital [16]
Microalbuminuria	1892.61 (217,650)	Ministry of Health, Labour and Welfare [19]
Macroalbuminuria	3256.41 (374,487)	Ministry of Health, Labour and Welfare [19]
ESRD^a^	6564.92 (754,966)	Ministry of Health, Labour and Welfare [19]
Cardiovascular events from diabetes	3587.30 (412,540)	Dentsu Digital [16]
Dialysis	41,739.13 (4,800,000)	Ministry of Health, Labour and Welfare [4]
CGM^b^ app	2,827.83 (3,25,200)	Ministry of Health, Labour and Welfare [19], Sakamaki et al [24]

^a^ESRD: end-stage renal disease.

^b^CGM: continuous glucose monitoring.

### Sensitivity Analysis

To confirm the robustness of our model, we carried out sensitivity analyses of stochastic transitions, utility values, and medical costs. The values for the sensitivity analysis of stochastic transitions were taken from the literature, if standard deviations were available, and were assigned from 0.5-fold to 2.0-fold if not. The values in the sensitivity analyses of both medical costs and utility values were a uniform ±10%.

### Calculation of the Incremental Cost-Effectiveness Ratio

We investigated the effectiveness of the use of a CGM app by calculating its incremental cost-effectiveness ratio (ICER). The ICER is obtained by dividing the difference between the medical cost of the medical technology under evaluation and that of the conventional technology (Δ Cost) by the difference between the utility value of the medical technology under evaluation and that of the conventional technology (Δ Effect).

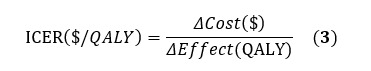



### Investigation of the Validity of the Markov Model

We investigated the validity of our Markov model from two aspects. The first was its internal validity, in which we considered it from the perspectives of whether it fully expressed the natural progression of the states concerned and whether the parameters used were appropriate. The second was its external validity, which we investigated by replacing the clinical data used in the model with other data and examining whether the estimated values thus obtained were appropriate. We picked up complications associated with DM, particularly the medical expenditure of a complication. In the process of model construction and selection of the parameters, the model overview and each parameter were reviewed by a cardiology specialist. At the same time, we adopted the values of the parameters from previous studies conducted in Japan. These operations guaranteed the validity of our Markov model, and we judged it could concisely express the progression of the states and the difference of medical expenditure derived from type 2 DM.

## Results

### Medical Costs

The total medical costs for patients undergoing insulin therapy were US $50,417,581,024 over 20 years with the use of a CGM app and US $47,817,427,894 over 20 years without app use, an increase of US $2,600,153,130 over 20 years. The increases in utility value, ICER, and QALY over 20 years were 78,699, US $33,039/QALY, and 0.11 QALY per person, respectively.

In terms of the medical costs for each patient state, CGM app use increased the costs (over 20 years) of insulin therapy (US $3,503,535,847), macroalbuminuria (US $20,981,856), and dialysis (US $1,465,525), whereas it decreased the cost (over 20 years) of microalbuminuria (US $842,698,713), ESRD (US $4,280,039), and cardiovascular disease (US $78,851,346). In terms of changes in patient numbers, the number of patients undergoing insulin therapy increased by 21,649 people over 20 years, and the number with macroalbuminuria increased by 830 people over 20 years, whereas the number of patients with microalbuminuria decreased by 13,547 people over 20 years, with ESRD by 22 people over 20 years, undergoing dialysis by 23 people over 20 years, and with cardiovascular disease by 3357 people over 20 years. There were 5529 fewer deaths over 20 years.

### Sensitivity Analyses

**Figure 2:**
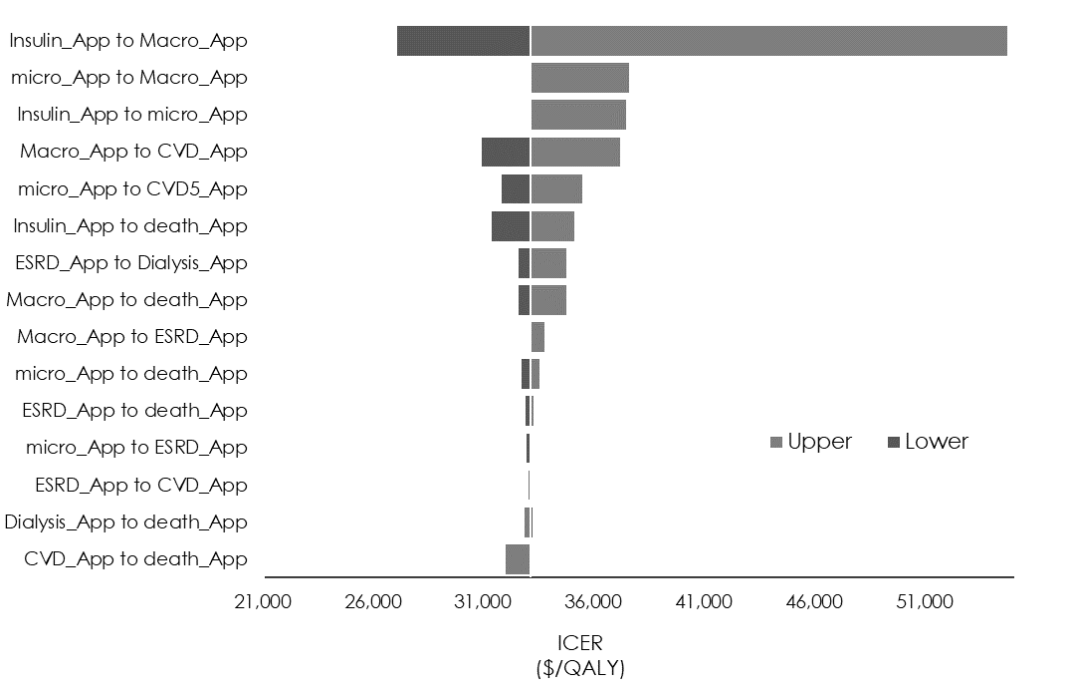
Sensitivity analysis of the incremental cost-effectiveness ratio (ICER) using transition probabilities. CVD: cardiovascular disease; ESRD: end-stage renal disease; QALY: quality-adjusted life year.

**Figure 3:**
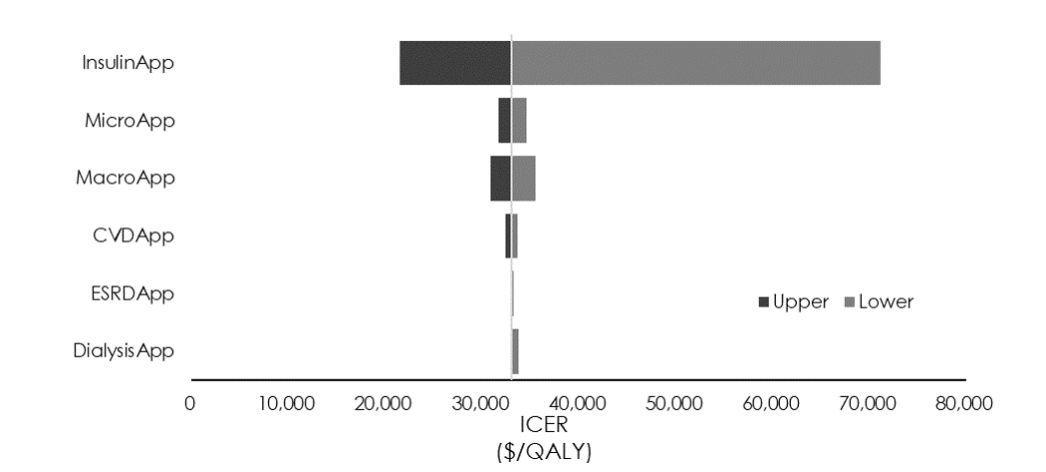
Sensitivity analysis of the incremental cost-effectiveness ratio (ICER) using utility values. CVD: cardiovascular disease; ESRD: end-stage renal disease; QALY: quality-adjusted life year.

**Figure 4:**
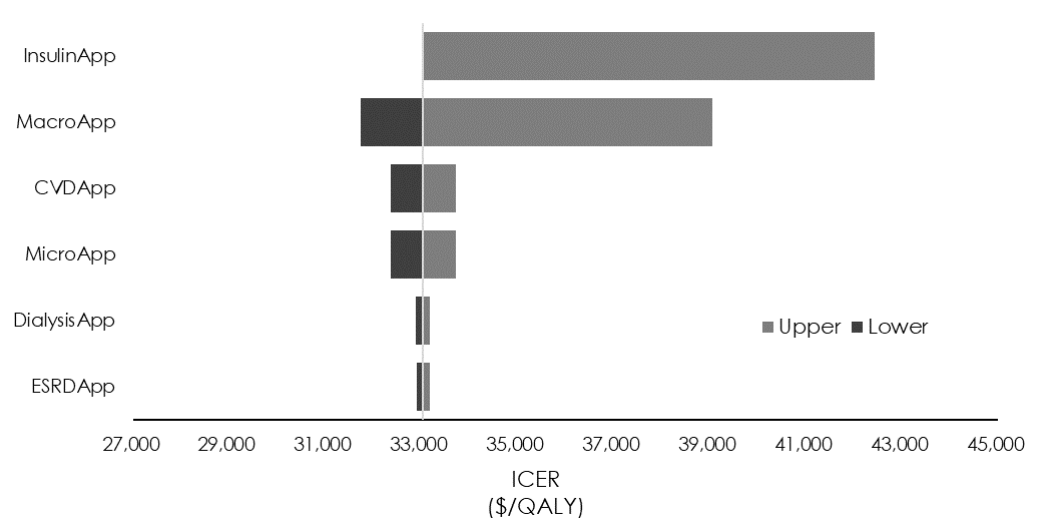
Sensitivity analysis of the incremental cost-effectiveness ratio (ICER) using medical fees. CVD: cardiovascular disease; ESRD: end-stage renal disease; QALY: quality-adjusted life year.

The sensitivity analysis of transition probabilities showed that the minimum and maximum ICERs (US $26,989/QALY and US $54,650/QALY) were obtained when the transition probability from insulin therapy to macroalbuminuria was 0.5 times or 2 times the standard value (1.24% and 5.35%, respectively; Figure 2). The sensitivity analysis of utility values showed that a variation of ±5% in the utility value of insulin therapy caused the ICER to vary from US $21,516/QALY to US $71,142/QALY (Figure 3). Finally, the sensitivity analysis of medical costs showed that the maximum variation occurred when the medical cost of insulin therapy was increased by 10% (US $42,436/QALY) and the medical cost of macroalbuminuria was decreased by 10% (US $31,732/QALY; Figure 4) We carried out sensitivity analyses to investigate the effect of variations in the CGM app use model parameters (transition probabilities, utility values, and medical costs) on the ICER. First, the results of the sensitivity analysis of transition probabilities showed that the ICER for transitioning from insulin therapy to macroalbuminuria was US $54,650/QALY. Although this is below the ICER used by the WHO [29], it greatly exceeds the value of US $43,478 (5 million yen) per QALY used by the Central Social Insurance Medical Council. The same trend was also evident for the second and third highest ICERs, which were seen when the transition probabilities from insulin therapy to microalbuminuria and from microalbuminuria to macroalbuminuria, respectively, were increased. From the ICER perspective, the introduction of CGM apps for patients who develop diabetic nephropathy and whose condition progresses rapidly should be viewed with caution. Next, the results of the sensitivity analysis of utility values showed that a reduction in the utility value of insulin therapy dramatically increased the ICER (to US $71,142/QALY). However, as the utility value for insulin therapy used in this study for patients using a CGM app was similar to that for patients not using such an app, the introduction of CGM apps is unlikely to cause the utility value to decline, and it is more likely that a rise in the utility value would decrease the ICER. Increasing the utility value by 10% may also reduce the ICER to US $21,516/QALY. With respect to the effect of a decrease in the utility value of microalbuminuria, which exhibited a high ICER, the value of US $35,517/QALY was well below the value of US $43,478/QALY used by the Central Social Insurance Medical Council, suggesting that it did not correspond to a factor causing major variation in the ICER obtained in this study. Finally, the sensitivity analysis of medical costs showed that an increase of 10% in the cost of insulin therapy increased the ICER from US $33,039/QALY to US $42,436/QALY (increase of US $9397/QALY), but this was very close to the value used by the Central Social Insurance Medical Council (US $43,478/QALY), suggesting that the introduction of CGM apps should be considered even if the cost of insulin therapy and other medical costs were to increase.

The WHO suggests that the ICER for the introduction of a new medical technology should be no more than three times GDP per capita [32]. Applied to the GDP of Japan, this figure would be US $116,649/QALY (as of 2016). The ICER used by the Central Social Insurance Medical Council is US $43,478/QALY (5,000,000 yen/QALY), less than one-quarter of the value proposed by the WHO. The ICER obtained in this study (US $33,039/QALY) is well below either of these two thresholds. In comparison, cost-effectiveness analyses of retrospective CGM (in type 1 DM patients) have reported ICERs between US $41,000/QALY and US $99,000/QALY [33-35]. For example, a cost-effectiveness analysis of self-injection compared with CGM plus intensified insulin therapy found that, over a period of 33 years, the ICER for CGM plus intensified insulin therapy was US US $45,033/QALY [32]. The ICER for the introduction of CGM apps that we found in our study (US $33,039/QALY) was thus both lower than the ICER for the conventional method of retrospective CGM and the ICERs suggested by the WHO and the Central Social Insurance Medical Council (US $116,649/QALY and US $43,478/QALY, respectively), indicating that the introduction of this medical technology should be considered in Japan.

## Discussion

In this study, we used a Markov model to calculate the ICER of the use of a CGM app to investigate the cost-effectiveness of such apps for patients receiving insulin treatment and calculated that the ICER was US $33,039/QALY.

### Internal and External Validity of the Model

The model used in this study was developed around the medical costs for patients undergoing insulin treatment with a focus on diabetic nephropathy, dialysis, and cardiovascular disease, all of which entail high medical costs. As far as possible, the values assigned to the parameters were obtained from publications describing patients of similar ages and backgrounds. The transition probability values and utility values for patients using a CGM app were basically taken from clinical data on intensified insulin therapy with the aim of avoiding any overestimation of the effect achieved by the introduction of CGM apps, and although the range of variation in the parameters as a result of the introduction of CGM apps is currently unclear, this was assured by sensitivity analysis. For our model in this study, we chose diseases with a major effect on medical costs and for which data on each of the parameters were available; for this reason, the model did not include neuropathy and retinopathy, two of the three major complications of type 2 DM. Because we were unable to obtain sufficient data on the proportion of patients whose diseases improved, this was not reflected in our model. On these two points, the model must be revised and revalidated as and when usable clinical data are published in the future.

In terms of the external validity of the model, a comparison of the transition probability for diabetic nephropathy used in this study and the prevalence reported by Yokoyama *et al [36]* showed that the prevalence of nephropathy in our study was higher until year 18 of type 2 DM, but that subsequently it was lower. One reason may have been that the prevalence of diabetic nephropathy is known to increase in patients who have had type 2 DM for longer [36], and the patients in the study by Yokoyama *et al* [36] were aged ≤30 years, whereas the data used in our study came from patients aged approximately 50 years. The difference in the transition probability caused by this age difference meant that the patients in our study may have transitioned to the state of death earlier than those studied by Yokoyama *et al *[36]. Although the clinical data were obtained from patients with diverse attributes, including some who had already developed complications, for the patients simulated in this study, the analysis started from the state of receiving insulin therapy alone, and this may have been another reason for the differences in prevalence.

The reported incidences of cardiovascular events induced by type 2 DM include those obtained from the Hisayama Study (5/1000 people/year) [37] and the Japan Diabetes Complications Study (14.7-17.4/1000 people/year) [38], and despite the differences in patient attributes, we obtained a similar rate in our study (11.0/1000 people/year over 20 years). Evidence from cohort studies will be required for further internal validation of the state of cardiovascular events. For dialysis, according to materials published by the Japan Society for Dialysis Therapy [18], diabetic nephropathy was the reason for the introduction of dialysis in 43.2% of patients who started dialysis in 2015. A calculation of the proportion of the 3,166,000 type 2 DM patients who were undergoing insulin therapy in 2015 and started on dialysis for diabetic nephropathy who were aged 70-75 years found that they constituted 24.18% of such patients, and in our model, the 1.20 million patients undergoing insulin treatment who were aged 70 years (after 20 years) also accounted for 25.16% of patients, a very similar figure. Other clinical data tend to compare data from patients with different attributes, and their evaluation is dependent on the data provided. Considered from the viewpoint of medical costs, the costs of treating type 2 DM, including insulin therapy, published by the MHLW in its 2016 summary of national medical costs (for patients aged 45-65 years, $2.7 billion yen for type 2 DM and $1.4 billion yen for cardiovascular disease) were higher than the medical costs for patients receiving insulin therapy estimated in this study (for patients age 60 years, approximately 2.5 billion yen), but the two were broadly consistent. To validate our model, comparisons with long-term cohort follow-up data are required, but the only method available is to compare the model's estimated values with the small number of cases reported in Japan; therefore, there is room for further analysis of the validation results.

### Limitations and Topics for Further Investigation

This study has several limitations. One of the limitations is the problem of assigning transition probabilities, as shown in the results of the investigation of the model's external validity. The patients in this study were 50 years old, and over a 20-year analysis period, the probability of developing complications would change rapidly as they aged, potentially generating errors compared with the actual numbers of patients over longer analysis periods. To bridge this gap with reality, it will be necessary to divide the analysis period into shorter periods and assign different transition probabilities for the different periods.

Second, we could not consider unmeasured values because this analysis was based on a simulation. There is no evidence that the assumed value should be due to a lack of data, such as the proportion of patients whose diseases improved after using the CGM app.

This study assumed that CGM apps offer the advantages of enabling patients to control their own blood glucose levels by monitoring them in real time and share this information with medical institutions and family members. Although the economic evaluation itself is likely not new, this simulation method has not been applied to CGM apps yet, as far as we were able to determine. Analysis of the cost-effectiveness of the second of these advantages, that of sharing blood glucose level information, is a topic for further investigation.

The present study is limited by a lack of considering the effect by population aging, because we employed transition probabilities as a constant in the construction of the Markov model. For instance, it is assumed that the per capita medical cost increases as the population ages, but it was not possible to consider the decrease in the number of patients due to the rise in mortality. Although this could be overcome in future studies, the present study was unable to incorporate this consideration.

Finally, we assumed that the effect of CGM app use on type 2 DM is the same as intensified insulin therapy. At this point, there is no evidence to support this assumption. Therefore, we could only perform a simulation analysis based on some assumptions. To measure cost-effectiveness with greater accuracy, further research or accumulation of real-world data about app use is required.

### Conclusion

We calculated that the ICER of the introduction of CGM apps for type 2 DM patients was $33,039/QALY, and a comparison with ICERs reported in previous studies and by independent organizations indicated that this is a medical technology worthy of consideration.

## Abbreviations

CGM: continuous glucose monitoring
CVD: cardiovascular disease
DM: diabetes mellitus
ESRD: end-stage renal disease
ICER: incremental cost-effectiveness ratio
MHLW: Ministry of Health, Labour and Welfare
QALY: quality-adjusted life year
WHO: World Health Organization
